# Obsessive-compulsive disorders as a result of traumatic brain injuries in middle age people

**DOI:** 10.1192/j.eurpsy.2026.11599

**Published:** 2026-07-31

**Authors:** N. Syrmos

**Affiliations:** Aristotle University, Thessaloniki, Greece

## Abstract

**Introduction:**

Introduction- Traumatic brain injury neuropsychiatric results are important causes of morbidity. Among of theme are the obsessive-compulsive disorders (OCD).

**Objectives:**

Aim-Aim of this study is to present cases with obsessive-compulsive disorders as a result of traumatic brain injuries in middle age people

**Methods:**

Material-Methods-10 patients are presented.7 males (70%) and 3 females (30%) Range of age between 50-65 years old. All of theme (10-100%) were evaluated, with clinical and radiological exam(ct,mri) plus diagnostic tests for Obsessive-compulsive disorders

**Results:**

Results-Good results in 8 cases (80%) and moderate in 2 (20%). Collaboration between various medical disciplines is essential in order to achive optimal results and clarify the theory,the etiology, and treatment effectiveness.

**Conclusions:**

Conclusion-Premorbid status can be obtained by structured psychiatric interviews, and traumatic brain lesions can be defined with advanced neuroimaging techniques

**Disclosure of Interest:**

None Declared

